# Current News

**Published:** 1904-09

**Authors:** 


### Current News.

### MISSISSIPPI VALLEY MEDICAL ASSOCIATION.

The thirtieth annual session of the Mississippi Valley Medical Association will be held at Cincinnati, Ohio, October 11, 12, and 13, 1904, under the presidency of Dr. Hugh T. Patrick, of Chicago. The head-quarters and meeting places will be at the Grand Hotel.

The annual orations will be delivered by Dr. William J. Mayo, of Rochester, Minn., in Surgery, and Dr. C. Travis Drennen, of Hot Springs, Ark., in Medicine.

Request for places upon the programme, or information in regard to the meeting, can be had by addressing the Secretary, Dr. Henry Enos Tuley, Louisville, Ky., or the Assistant Secretary, Dr. S. C. Stanton, Masonic Temple, Chicago, Ill.

The usual railroad rates will be in effect.

Henry Enos Tuley,
*Secretary.*

### HARVARD DENTAL ALUMNI ASSOCIATION.

The following officers were elected June 27, 1904, at the thirty-third annual meeting of the Harvard Dental Alumni Association, held at Young's Hotel, Boston:

President, Harry S. Parsons, '92, Boston; Vice-President, Ned A. Stanley, '84, New Bedford; Secretary, Waldo E. Boardman, '86, Boston; Treasurer, Harold De W. Cross, '96, Nashua, N. H.

*Executive Committee.*—Waldo E. Boardman, '86, chairman, *ex officio,* Boston; Samuel T. Elliott, '01 (for one year), Boston; Walter A. Davis, '01 (for two years), Boston.

The council is composed of the above-named officers.

Waldo E. Boardman, '86,
*Secretary.*

---

### NORTHERN INDIANA DENTAL SOCIETY.

The date of our next annual meeting, to be held at Huntington, Ind., has been postponed to October 18 and 19, 1904.

A programme of unusual interest has been completed, a synopsis of which will be announced in the next issue of this magazine.

Don't forget to read it.

Otto U. King,
*Secretary.*

King Building, Huntington, Ind.

---

### SOUTHWESTERN IOWA DENTAL SOCIETY.

The eighth annual meeting of the Southwestern Iowa Dental Society will be held October 11 and 12, 1904, at Osceola, Iowa.

J. A. West,
*Secretary.*

Creston, Iowa.

